# A Systematic Review of Methods and Criteria Standard Proposal for the Use of Principal Component Analysis in Team’s Sports Science

**DOI:** 10.3390/ijerph17238712

**Published:** 2020-11-24

**Authors:** Daniel Rojas-Valverde, José Pino-Ortega, Carlos D. Gómez-Carmona, Markel Rico-González

**Affiliations:** 1 Centro de Investigación y Diagnóstico en Salud y Deporte (CIDISAD), Escuela de Ciencias del Movimiento Humano y Calidad de Vida (CIEMHCAVI), Universidad Nacional, Heredia 86-3000, Costa Rica; 2 Grupo de Avances en el Entrenamiento Deportivo y Acondicionamiento Físico (GAEDAF), Facultad Ciencias del Deporte, Universidad de Extremadura, 10071 Cáceres, Spain; 3 Department of Physical Activity and Sport Sciences, International Excellence Campus “Mare Nostrum”, Faculty of Sports Sciences, University of Murcia, 30720 San Javier, Spain; 4 Biovetmed & Sportsci Research Group, University of Murcia, 30100 Murcia, Spain; 5 Research Group in Optimization of Training and Sports Performance (GOERD), Department of Didactics of Music, Plastic and Body Expression, Sports Science Faculty, University of Extremadura, 10071 Caceres, Spain; cdgomezcarmona@unex.es; 6 Departament of Physical Education and Sport, University of the Basque Country, UPV-EHU, Lasarte 71, 01007 Vitoria-Gasteiz, Spain

**Keywords:** PCA, factor analysis, statistic, big data

## Abstract

The availability of critical information about training and competition is fundamental on performance. Principal components analysis (PCA) is widely used in sports as a multivariate technique to manage big data from different technological assessments. This systematic review aimed to explore the methods reported and statistical criteria used in team’s sports science and to propose a criteria standard to report PCA in further applications. A systematic electronic search was developed through four electronic databases and a total of 45 studies were included in the review for final analysis. Inclusion criteria: (i) of the studies we looked at, 22.22% performed factorability processes with different retention criteria (*r* > 0.4–0.7); (ii) 21 studies confirmed sample adequacy using Kaiser-Meyer-Olkim (KMO > 5–8) and 22 reported Bartlett’s sphericity; (iii) factor retention was considered if eigenvalues >1–1.5 (*n* = 29); (iv) 23 studies reported loading retention (>0.4–0.7); and (v) used VariMax as the rotation method (48.9%). A lack of consistency and serious voids in reporting of essential methodological information was found. Twenty-one items were selected to provide a standard quality criterion to report methods sections when using PCA. These evidence-based criteria will lead to a better understanding and applicability of the results and future study replications.

## 1. Introduction

The ability to assess performance is one of the primary roles of sports scientists and analysts [1]. Consequently, the use of notational analysis in sport has become an essential tool to identify critical patterns and events that could lead to a successful outcome. That is why the sports scientist and performance analyst figure has recovered particular relevance [2,3,4], providing information that could enhance the observation, recall, analysis, and feedback quality of athletes, trainers and coaches. In this sense, some statistical techniques allow the information collected in areas of competence—suchas tactical and technical behavior, adaptations, and acute and chronic responses at a physical and physiological level—to be analyzed simply and reported to head coaches in an effective, efficient and objective way. This in turn allows for quick decision making which is crucial for the maintenance and improvement of sports performance [4]. The information resulting from these kinds of analyses could lead to new training techniques, match strategies, recovery protocols, injury prevention programs, and other decisions based on daily monitored data.

The development of new technology has led to the availability of a high amount of data obtained during training and competition. Some devices such as electronic performance, tracking systems, and microelectromechanical systems have allowed up to a thousand data per second in an amount of up to 400 variables depending on the technology used [5]. These data sets or combinations of data sets whose volume, complexity, variability, and speed of growth hinder their capture, management, processing, or analysis using conventional technologies and tools is called big data [6]. In the practical setting, where the fast evaluation of training/competition loads is necessary to assess performance and inform exercise prescription, big data should be reduced. In sport, this data management has been done through multivariate data analysis techniques such as exploratory factor analysis (EFA) to explain many measured variables using a smaller number of extracted factors [7,8,9]. These variables can then be used in the following analysis, such as cluster or regression analysis, to gain a better explanation of some sport behaviors and adaptations [10,11]. Despite the several factor extraction methods (e.g., maximum likelihood, alpha factoring, generalized least-products, unweighted least-squares, or principal axis factoring), principal component analysis (PCA) is one of the most used statistical techniques in sport [12].

PCA is defined as a data reduction technique usually used in sports to identify key performance indicators. Coaches, trainers, and athletes tend to select the variables to monitor performance according to their professional experience and other athlete’s information (evidence-based or not), but with the potential of excluding essential variables [13]. This justifies the need for the use of objective methods of selecting these critical variables. In this case, the PCA technique allows sports scientists and researchers to select and extract the variables that explain a high percentage (>70%) of the total variance of a certain amount of data during a specific time window (e.g., session, micro cycle, season). The potential of reducing a big group of correlated variables in a series of uncorrelated ones allows for simplifying of athletes and team behavior [4]. PCA has been used in different sports to extract the most relevant physical, physiological, technical, or tactical variables that explain performance. Researchers have explored the use of this statistical model to understand the behavior of sports such as rugby [9,14], soccer [15], and basketball [16]. However, the studies using PCA usually do not provide sufficient data to allow researchers and stakeholders to make interpretations or understand how and why the final results were obtained.

Considering the inherent subjectivity of this factor analysis process, there may be some methodological issues when the data is analyzed and interpreted [17]. Some of these methodological decisions may result in different outcomes [18], leading to possible erroneous interpretations of the analysis and potentially wrong conclusions [19]. In a sport setting, these mistakes can provoke some faulty programming and load prescription and in-field decisions that could impact global performance. Therefore, it is crucial to explore and systematize the methodological choices and criteria for data mining selection, the suitability of the data set, factor extraction, factor retention rules, and factor rotations. Besides, principal component extraction criteria and final interpretation and labeling of the analysis outcomes should be reported [12]. Thus, this systematic review aimed explore the methods reported and statistical criteria used in team sports science and b) to propose a criteria standard to report PCA in further applications.

## 2. Materials and Methods

**Figure 1:**
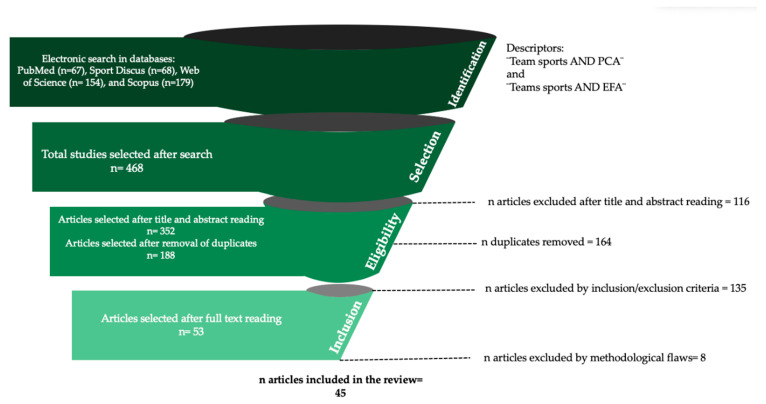
Flow diagram of the study’s identification selection and inclusion.

This systematic review was conducted based on the principles of the Preferred Reporting Items for Systematic Reviews and Meta-Analyses (PRISMA) guidelines [20]. After compiling the studies, they were classified by year, identifying those that met the inclusion criteria for final selection and extraction (see Figure 1). Two authors independently reviewed studies for their eligibility. Discrepancies between authors were resolved using consensus. Given the study type (i.e., systematic review article), ethical approval was not necessary.

### 2.1. Information Sources and Search Strategy

An electronic systematic review of literature search was computed through four different databases: PubMed (*n* = 67), Web of Science (*n* = 154), SPORTDiscus (*n* = 68) and Scopus (*n* = 179). This search was performed on November 1st, 2019, before 9:00 a.m., to identify studies investigating PCA use in a team sport. The authors did not discriminate by journal names or manuscript authors. The search strategy used the combination of terms related to population (team sport, soccer, football, basketball, rugby, hockey, futsal, handball) and intervention (principal component analysis and exploratory factor analysis). The search was made using combinations of the keywords using the Boolean operators “and” (inter-group Boolean operator) and “or” (intra-group Boolean operator, only for the second). All references were extracted and imported into an open-source research tool (5.0.64, Zotero, Fairfax, VA, USA) to systematize studies.

### 2.2. Studies Selection

The following inclusion criteria were considered. Studies containing keywords in the title or abstract, and studies published from 2000 to 2020. A single author accessed the original primary data from the studies (title, authors, date, and database) to an Excel spreadsheet (Microsoft Excel, Microsoft, Redmond, DC, USA) and removed the duplicate records. After duplicate removal, two authors contrasted results independently considering inclusion and exclusion criteria. The authors were not blinded to the title or authors of the publications. Any disagreements on the final inclusion or exclusion decisions were solved through consensus when screening and excluding studies. Abstracts, conference papers, and other reports were not included. Documents published in the English language were included, and other languages (e.g., Spanish, German, and Italian) were included if a translation could be performed.

### 2.3. Data Collecting

Two different authors performed the studies’ selection and extraction following the PRISMA protocol (see Figure 1). Specific exclusion criteria were used to discard studies. This included low quality, irrelevance to the primary purpose of this systematic review, language limitations, different evaluation methods, full text not available, book chapters, abstracts, studies involving factors other than team sports, no use of technology tools assessment methods, no competitive, elite, or professional players involved and severe lack of information (e.g., no sports specification, no participant characteristics, no PCA results or variance were reported). The protocol followed for selecting the studies was as follows: (i) identification of potential studies; (ii). elimination of duplicates; (iii)title, abstract, and year analysis; (iv). quality of method and relevance with the review’s objective analyzed; and (v) selected studies explored in full text. Studies with a lack of information were excluded.

**Figure 2:**
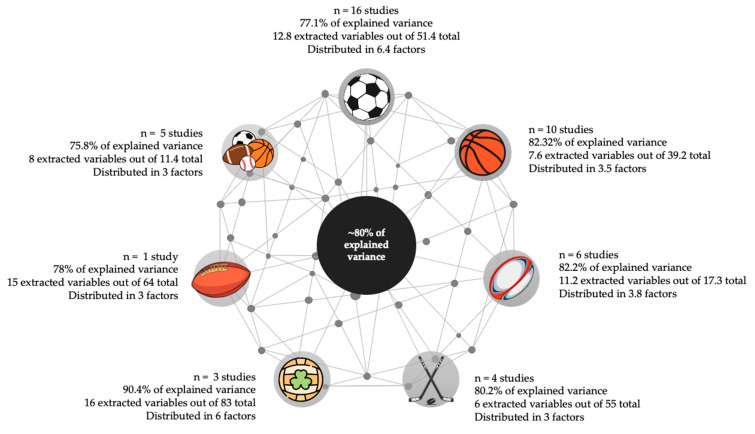
Sample characteristics with variable extraction and analysis outcomes. Data are presented in the mean of total studies per sport.

The methodological approach involved analyzing the criteria used to perform exploratory analysis considering retention loading criteria, data suitability testing, extraction method used, factor and loading retention criteria selected, and rotation method if performed (see Table 1). The EFA outcomes were resumed considering number of articles in each sport (discipline), sample size, number of extracted factors, percentage of variances explained and number of variables extracted (see Figure 2).

## 3. Results

Of the 468 papers initially identified from the databases, 116 were excluded after considering the title, abstract, and year of publication. Once the duplicates were removed, a total of 188 articles were analyzed, considering exclusion and inclusion criteria. From those remaining studies, only 53 studies were read in full text, and due to lack of vital information, eight studies were excluded. Table 1 shows the compilation of analysis for 45 selected studies included in this systematic review and describes the main methodological aspects used in each protocol.

### 3.1. Sample Characteristics

The studies selected performed PCA in sports like soccer, basketball, rugby, hockey, Gaelic football, Australian football, and other combined sports (see Figure 2). The PCA was selected to explore physical performance variables, technical and tactical variables, locomotion and physical load variables, and biomechanical and biochemical results. Explaining 80 ± 0.14% of the total variance of data sets, from 36.59 ± 80.79 variables analyzed by PCA were selected 9.12 ± 5.73 variables, that are distributed in 3.9 ± 2.53 factors.

### 3.2. Methodological Criteria Used

The following section discusses the information provided and the information missing in PCA reporting of the articles published in sports. All of the studies (100%, *n* = 45) selected PCA as the exploratory factor analysis (EFA). From those articles included in this systematic review only 22.22% (*n* = 10) reported to perform an exploration of correlation matrix of variables prior running PCA analysis. The retention criteria used for variable selection in this previous step were *r* > 0.4 = 4.44% (*n* = 2), *r* > 5 = 8.9% (*n* = 4), *r* > 5.5 = 2.22% (*n* = 1) and *r* > 0.7 = 6.7% (*n* = 3) (Table 1).

Sample adequacy criteria was confirmed using Kaiser-Meyer-Olkim (KMO) suitability test and sphericity was explored using Bartlettߣs test. KMO reported values were >5 = 17.8% (*n* = 8), >6 = 4.44% (*n* = 2), >7 = 17.8% (*n* = 8) and >8 = 6.7% (*n* = 3). A total of 48.9% (*n* = 22) of the studies reported Bartlett´s Sphericity test suitability confirmation (Table 1).

Factor retention was considered if eigenvalues were >1 in 62.22% (*n* = 28) of studies and >1.5 in 2.22% studies (*n* = 1). The following loading retention criteria were used: >0.4 = 4.44% (*n* = 2), >0.6 = 20% (*n* = 9), >0.65 = 2.22% (*n* = 1) and >0.7 = 17.8% (*n* = 8). Only 6.7% (*n* = 3) of articles reported a criterion when cross-loading was found, and the highest loading was selected. The preferred rotation method was orthogonal VariMax rotation in 48.9% of cases (*n* = 22), followed by Direct Oblimin used by 4.44% of studies (*n* = 2) and a nonorthogonal rotation method used by 2.22% of articles (*n* = 1) (Table 1).

## 4. Discussion

The main finding was a lack of consistency between articles and serious voids in the methodology sections’ information. A total of 21 methodological requirements were identified as crucial quality criteria to report both methods and results when using PCA as a data reduction technique.

Commonly, PCA can result in several number solutions and outcomes based on the researchers’ subjective decisions. When conducting PCA, these methodological decisions could result in different outcomes depending on the aims of the research, even though these decisions may be critical for the practical applications of the results obtained. Commonly, the majority of studies did not provide enough information to allow medical staff, coaches, athletes, and sports scientists to make independent interpretations or at least understand how the final results were obtained.

To perform EFA properly, it is necessary to follow specific guidelines that will entail high-quality results [10,12]. The first step is to examine the correlation between variables to extract uncorrelated variables considered in the EFA. This initial process is known as the factorability of *r* [17]. Some correlation coefficients have been proposed as a threshold to select variables. This systematic review found that only 22.2% of total studies reported correlation matrix inspection with a threshold of *r* > 5 = 8.9% and *r* > 0.7 = 6.7% as the preferred ones. In this regard, some authors have suggested that *r* > 5 is practically significant [62]. If factorability resulted in less than 0.3, this could be a clue that EFA might not be the appropriate statistical method [17,62].

Before the proper extraction of factors, some tests should be performed to assess the data’s suitability for factor analysis. The most common ones include KMO for sample adequacy and Bartlett’s test of sphericity. In this systematic review, KMO values were reported by 46.7% of the studies, and the most stated values were >5 and >7 in 17.8% of the cases each. When KMO > 5, the data set is considered suitable for EFA [62,63]. The Bartlett’s Test should be significant to be suitable [62,64]. In this study, only 48.9% of the articles reported this information.

After this suitability confirmation, the authors may report how the factors will be extracted. It was found that all scientists selected PCA as the preferred EFA in team sports analysis. This technique is considered one of the most useful and advantageous statistical methods for extracting the most representative variables of a data set [18] with a minimal loss of original data [65]. Considering that existing technology can give a large amount of data per second, this technique is fundamental to selecting those variables that could better explain team physical, technical, tactical, biomechanical, and workload-related variables as relevant information in decision making. This is fundamental because actual sports are required to collect, analyze, and present data as quick and straightforward as possible to the technical staff to achieve optimal performance [4].

Another consideration is the rotational method selected. Rotation maximizes high item loading and minimizes low item loadings, increasing simplicity, and interpretability [12]. VariMax orthogonal rotation is the most common technique used in EFA [66], and this is confirmed in 48.9% of the studies included in this systematic review.

Despite various rotation methods such as EquiMax, VariMax, QuartiMax, ProMax, and Direct Oblimin, they are equally useful to recover the underlying factor structure [67] but with particular differences. Orthogonal rotation methods (VariMax, QuartiMax, EquiMax) assume that the factors are uncorrelated, and in contrast, oblique rotation methods (e.g., Direct Oblimin, ProMax) assume the factors are correlated [68].

In sport science where the purpose is to reduce the number of variables that could explain the physiological, technical, tactical, and physical behavior of team sports players, the VariMax method could be the preferred technique due to some particularities. The VariMax method uses mathematical algorithms that maximize the high and low factor loadings while minimizing the mid-value factor loadings. It has been highlighted as the most widely used orthogonal rotation, considering that researchers can choose to represent factors as uncorrelated to meet some assumptions of a specific research purpose (e.g., multiple regression analysis that requires multicollinearity), although, in real settings, factors are usually correlated. Sports scientists usually perform a subsequent inferential analysis after performing PCA [16,48,50,60,61] which could be a reason to select VariMax over other methods.

The authors may also report what criteria will assist in determining how factors will be extracted after PCA. In this regard, for factor retention, the most used criterion is the Kaiser’s criteria, considering an eigenvalue >1 as a rule, this is deemed to be significant and constructs variables known as varifactors or loadings [69]. This criterion defined the number of components to be retained [63]. This is in line with the present study results in which Kaiser’s criterion was used in 62.2% of the cases. Some authors suggest a visual analysis of eigenvalues’ screen plot which is based on researcher judgment [17].

Additionally, loading retention criteria is needed to be informed. In this review, the most selected criteria were >0.6 and >0.7 in 20% and 17.8% of the studies, respectively. Values greater than 0.75 are usually considered strong; values from 0.5 to 0.75 moderate; and 0.3 to 0.49 are considered weak factor loadings [70]. It is assumed that loadings >0.7 could be used as selection criteria [62].

As evidenced, for serious quality management when using PCA, some specific methodological considerations should be considered. Studies may provide sufficient data to allow researchers and stakeholders to make interpretations and understand how and why the final results were obtained. This is why the authors propose a standard quality assessment criterion for evaluating and reporting PCA in team sport research (see Table 2). The authors have contrasted the actual evidence and the methodological and statistical standards and propose a 21 items survey that may guide future researchers to perform and report both methodological proceedings and the study´s outcomes. This survey must be completed, and the total score must be reported out of 21 items.

## 5. Conclusions

This systematic review found that sports science studies related to team sports usually lack methodological rigor when reporting principal component analysis. Less than 50% of articles did not state essential criteria for factorability or data suitability testing retention criteria of factors, loadings, and procedures when cross-loadings are found. Consequently, a 21 checklist was developed related to the methodological and results sections as a standard quality criteria recommendation when reporting PCA in team sports.

The information resumed in this systematic review allows recommending standard quality criteria for future studies based on reported methods and literature recommendations. This survey (see Table 2) will enable sport scientists and medical researchers to screen their methods and results sections to better report their selected criteria. This will lead to a better understanding and applicability of the results as well as future study replications.

## Figures and Tables

**Table 1:** Methodological criteria used on exploratory factor analysis in team sports.

Code	Reference	Sport	Factorability	Data Suitability Testing		Extraction Method	Retention Criteria			Rotation Method
			Retention Loading Criteria	Sample Adequacy Criteria	Sphericity Criteria		Factor	Loading	Cross-Loading	
1	Sampaio et al. [21]	Basketball	*r* > 0.5	KMO = 0.78	NR	PCA	>1	>0.4	NR	NR
2	Andrade et al. [22]	Basketball	NR	NR	Bartlett’s	PCA	>1	NR	NR	VariMax
3	Gómez et al. [23]	Soccer	*r* > 0.5	KMO = 0.65	NR	PCA	>1.5	>0.6	NR	VariMax
4	Ricotti et al. [24]	Soccer	NR	NR	NR	PCA	NR	NR	NR	NR
5	Liu [25]	Basketball	NR	NR	NR	PCA	NR	NR	NR	NR
6	Yin [26]	Basketball	NR	KMO = 0.8	Bartlett’s	PCA	NR	NR	NR	NR
7	Yin [27]	Basketball	NR	KMO = 0.8	Bartlett’s	PCA	NR	NR	NR	NR
8	Parrington et al. [28]	Australian Football	*r* > 0.4	KMO > 0.05	Bartlett’s	PCA	>1	>0.6–0.7	NR	NR
9	Laffaye & Tombleson [29]	Team sports	NR	NR	NR	PCA	>1	NR	NR	VariMax
10	Ra et al. [30]	Soccer	NR	NR	NR	PCA	NR	NR	NR	NR
11	Weaving et al. [14]	Rugby	NR	KMO = 0.5–0.75	Bartlett’s	PCA	>1	NR	NR	NR
12	Carpita et al. [31]	Soccer	NR	NR	NR	PCA	NR	NR	NR	NR
13	Arruda et al. [32]	Soccer	NR	NR	NR	PCA	NR	NR	NR	NR
14	Zago et al. [33]	Soccer	NR	NR	NR	PCA	>1	NR	NR	NR
15	Leiva & Amú-Ruiz [34]	Team Sports	NR	NR	NR	PCA	NR	NR	NR	NR
16	Torrents et al. [35]	Soccer	NR	NR	NR	PCA	>1	NR	NR	Direct Oblimin
17	Ric et al. [36]	Soccer	NR	NR	NR	PCA	>1	NR	NR	NR
18	Abdullah et al. [37]	Soccer	NR	KMO = 0.77	Bartlett’s	PCA	>1	>0.7	NR	VariMax
19	Abdullah et al. [38]	Soccer Hockey	*r* > 0.5	KMO = 0.65–0.69	Bartlett’s	PCA	>1	>0.6	NR	VariMax
20	Negra et al. [39]	Soccer and Handball	NR	KMO = NR	NR	PCA	>1	NR	NR	NR
21	Abdullah et al. [40]	Soccer	*r* > 0.5	KMO = 0.7–0.73	Bartlett’s	PCA	>1	>0.6	NR	VariMax
22	Los Arcos et al. [41]	Soccer	>0.55	NR	NR	PCA	>1	NR	NR	Nonorthogonal
23	Alias et al. [42]	Field hockey	NR	KMO = 055–0.58	Bartlett’s	PCA	>1	>0.7	NR	VariMax
24	Williams et al. [43]	Rugby	NR	KMO > 0.05	Bartlett’s	PCA	>1	>0.7	NR	VariMax
25	Weaving et al. [44]	Rugby	NR	KMO = 0.59	Bartlett’s	PCA	>1	>0.7	NR	VariMax
26	Maliki et al. [45]	Soccer	NR	KMO = 0.77	Bartlett’s	PCA	>1	NR	NR	VariMax
27	Razali et al. [46]	Hockey	NR	KMO = 0.54	Bartlett’s	PCA	NR	>0.7	NR	VariMax
28	Parmar et al. [9]	Rugby	NR	NR	NR	PCA	>1	NR	NR	VariMax
29	Henderson et al. [47]	Rugby	NR	NR	NR	PCA	NR	>0.4	NR	NR
30	Svilar, Castellano, Jukic, et al. [48]	Basketball	NR	KMO = 0.84–0.85	Bartlett’s	PCA	>1	>0.7	NR	VariMax
31	Teramoto et al. [49]	Basketball	NR	KMO = 0.78	Bartlett’s	PCA	>1	>0.6	NR	Direct Oblimin
32	Robbins et al. [50]	Ice hockey	NR	NR	NR	PCA	NR	NR	NR	NR
33	Floría et al. [51]	Basketball	NR	NR	NR	PCA	NR	NR	NR	NR
34	Maliki et al. [52]	Soccer	NR	KMO = NR	Bartlett’s	PCA	>1	>0.65	NR	VariMax
35	Weaving et al. [53]	Rugby	NR	NR	NR	PCA	>1	>0.7	NR	VariMax
36	Hilgemberg et al. [54]	Futsal, Handball, Basketball and Volleyball	NR	KMO = NR	Bartlett´s	PCA	NR	NR	NR	VariMax
37	Welch et al. [55]	Gaelic football	>0.4	NR	NR	PCA	NR	NR	NR	NR
38	Welch et al. [56]	Gaelic football	NR	NR	NR	PCA	NR	NR	NR	NR
39	Verheul et al. [57]	Team Sports	NR	NR	NR	PCA	NR	NR	NR	NR
40	Goncalves et al. [58]	Soccer	NR	KMO > 0.5	Bartlett’s	PCA	>1	>0.6	NR	VariMax
41	Casamichana et al. [15]	Soccer	NR	KMO = NR	Bartlett’s	PCA	>1	>0.7	NR	VariMax
42	Gamble et al. [59]	Gaelic football	NR	KMO = 0.73	NR	PCA	>1	NR	NR	VariMax
43	Pino-Ortega et al. [60]	Basketball	*r* > 0.7	KMO = 0.77	Bartlett’s	PCA	>1	>0.6	Highest Loading	VariMax
44	Oliva-Lozano et al. [10]	Soccer	*r* > 0.7	KMO = 0.78	Bartlett’s	PCA	>1	>0.6	Highest Loading	VariMax
45	Rojas-Valverde et al. [61]	Basketball	*r* > 0.7	KMO > 0.5	Bartlett’s	PCA	>1	>0.6	Highest Loading	VariMax

KMO = Kaiser-Meyer-Olkin, PCA = principal components analysis, NR = not reported.

**Table 2:** Standard quality criteria survey for studies reporting principal component analysis in sports.

Item	Reported	Non-Reported	Recommended Criteria
**Statistical Analysis Section**			
Was the initial number of variables reported?	Yes = 1	No = 0	-
Was the variable selection criterion reported based on sports characteristics?	Yes = 1	No = 0	-
Was a correlation matrix exploration (factorability) between variables performed?	Yes = 1	No = 0	-
Was a correlation retention loading criterion (factorability) reported?	Yes = 1	No = 0	Factorability > 5
Was the number of variables reported after correlation matrix exploration (factorability)?	Yes = 1	No = 0	-
Were the variables scaled and centered (if necessary)?	Yes = 1	No = 0	-
Was the data suitability (sample adequacy criteria) test performed?	Yes = 1	No = 0	KMO test
Was the data suitability (sample adequacy criteria) testing result reported?	Yes = 1	No = 0	KMO > 5
Was the data suitability (sphericity criteria) test performed?	Yes = 1	No = 0	Bartlett´s Test
Was the data suitability (sphericity criteria) testing result reported?	Yes = 1	No = 0	*p* < 0.05
Was the extraction method reported?	Yes = 1	No = 0	PCA
Were the retention criteria (factors) reported?	Yes = 1	No = 0	Eigenvalues > 1
Were the retention criteria (loadings) reported?	Yes = 1	No = 0	Loadings > 0.6—0.7
Was the cross-loading retention criterion reported?	Yes = 1	No = 0	Highest loading
Was the rotation method reported?	Yes = 1	No = 0	VariMax (may vary)
Was the post PCA following analysis reported?	Yes = 1	No = 0	-
Results section			
Was the number of factors extracted reported?	Yes = 1	No = 0	-
Was the final number of variables reported?	Yes = 1	No = 0	-
Were partial and total variance reported?	Yes = 1	No = 0	-
Was the percentage of total variance reported?	Yes = 1	No = 0	-
Were the eigenvalues reported?	Yes = 1	No = 0	-
Were the final variables selection reported?	Yes = 1	No = 0	-
Total punctuation	21	0	
